# An Unusual Case of Alveolar Rhabdomyosarcoma of the Neck in an Adult Patient

**DOI:** 10.7759/cureus.6745

**Published:** 2020-01-22

**Authors:** Mohammed Wazir, Akriti G Jain, Mohammed FaisalUddin, Daniel Tambunan

**Affiliations:** 1 Internal Medicine, Brigham and Women's Hospital, Boston, USA; 2 Internal Medicine, AdventHealth, Orlando, USA; 3 Internal Medicine, Deccan College of Medical Sciences, Hyderabad, IND; 4 Internal Medicine, Florida Hospital, Orlando, USA

**Keywords:** rhabdomyosarcoma, neck mass, tumor, adult rhabdomyosarcoma

## Abstract

Rhabdomyosarcoma is the most common soft tissue malignancy in adolescents. It is extremely rare for sarcomas to occur in patients more than 18 years of age. We present a case of rhabdomyosarcoma originating in skeletal muscles on the right side of the neck. Our case demonstrates the importance of considering an extensive differential diagnosis for a neck mass in adults. Our patient was diagnosed with alveolar variant of rhabdomyosarcoma and underwent chemotherapy and radiation therapy, but eventually due to recurrence opted for palliative therapy.

## Introduction

Rhabdomyosarcoma (RMS) is a tumor originating from skeletal muscle. It is the most common soft tissue sarcoma encountered in childhood and adolescence with highest incidence in the age group of one to four years. It is extremely rare for RMS to occur in a patient older than 18 years of age [1]. It is known to involve the head and neck region with orbits, paranasal sinuses, and cheek and neck being the most common affected sites in that region. Outcome in adults is very poor as the diagnosis is usually either missed or delayed due to rarity, incorrect diagnosis, and earlier spread to local and distant tissues. We present a rare case of right neck RMS in a 62-year-old male.

## Case presentation

**Figure 1:**
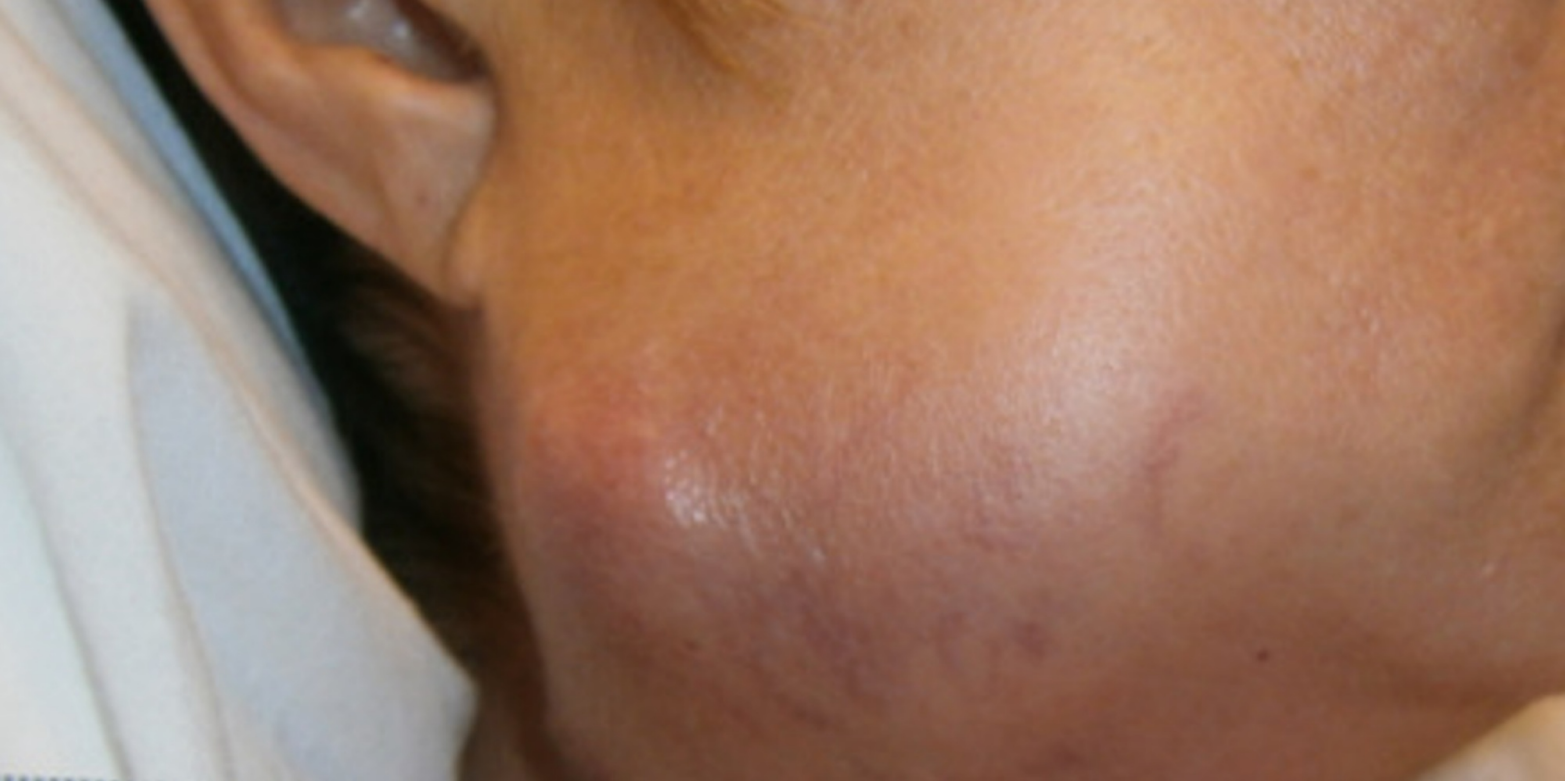
Enlarging right submandibular neck mass.

**Figure 2:**
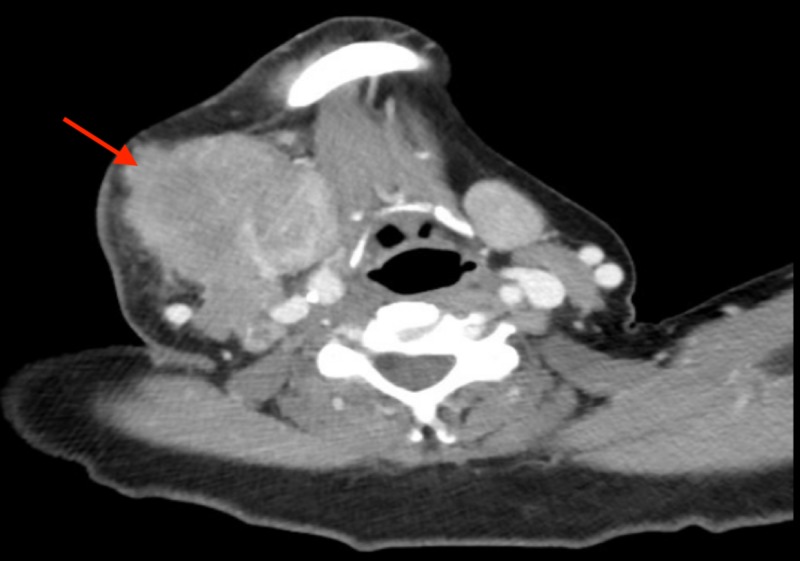
CT scan showing enlarging mass in the neck region.

**Figure 3:**
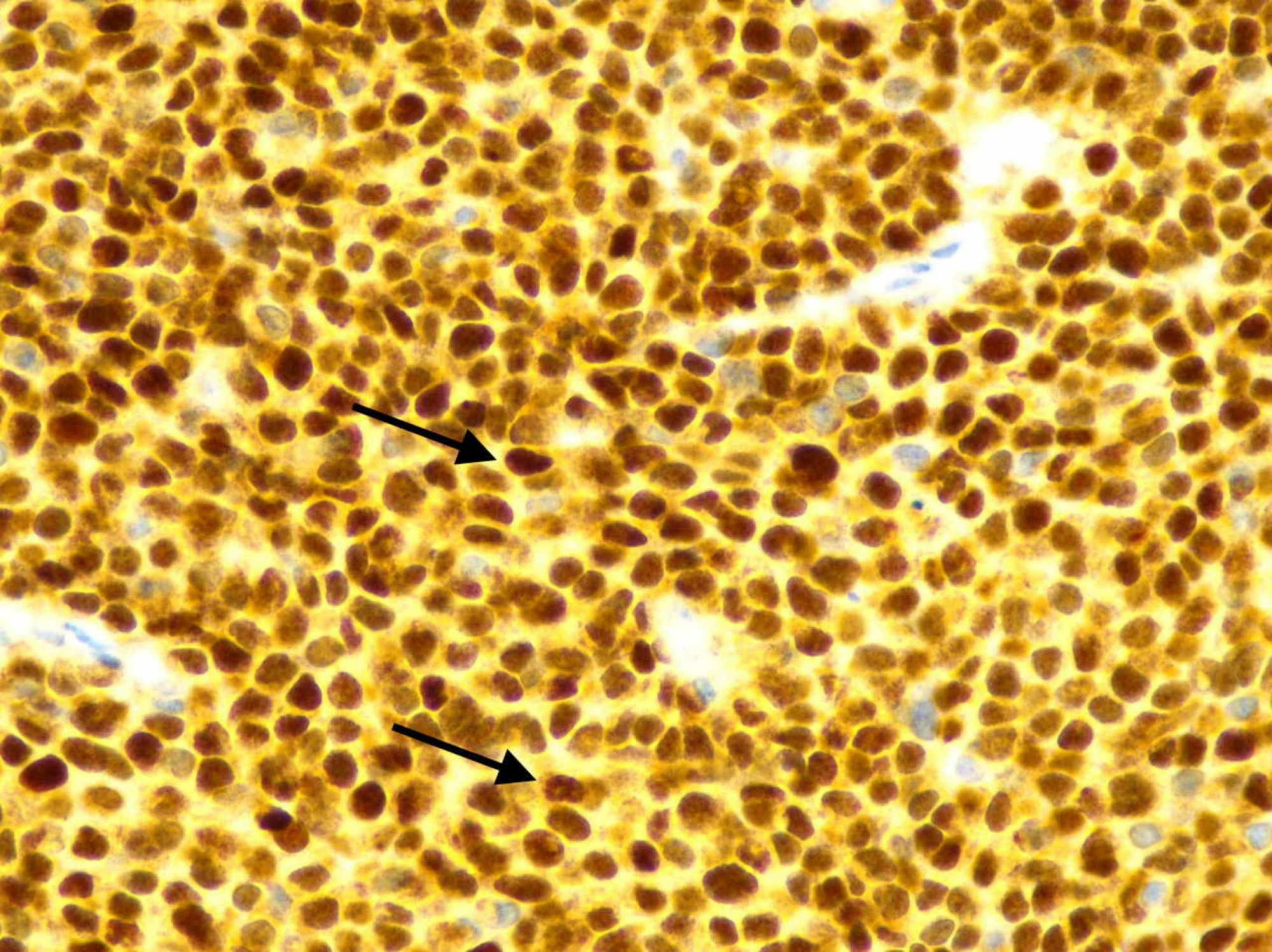
Biopsy of facial mass showing small blue cells (lymphocytes) with tumor positive for Myo D1, characteristic of rhabdomyosarcoma along with desmin positivity among other stains.

**Figure 4:**
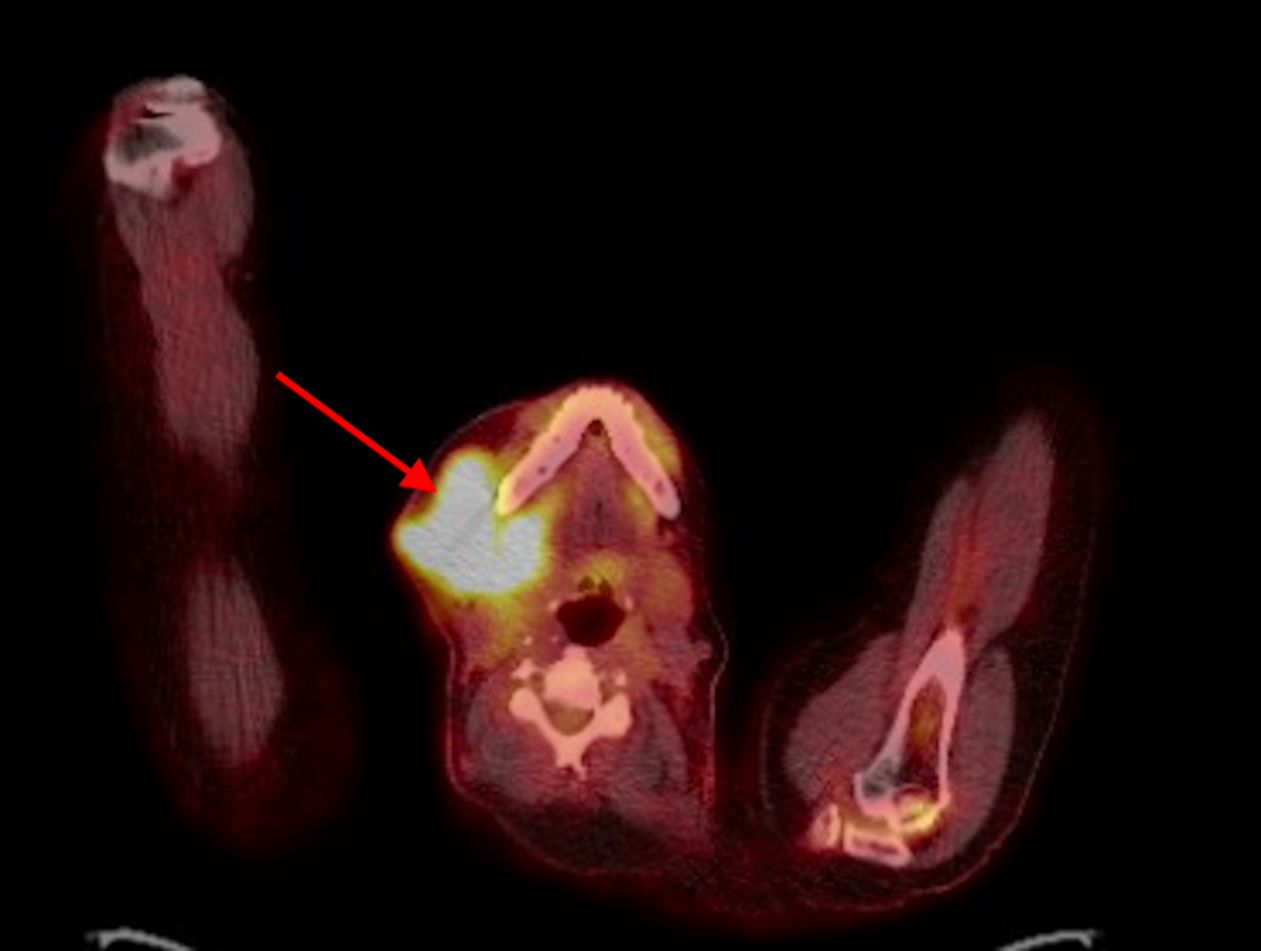
PET/CT showing the hypermetabolic right facial soft tissue mass (red arrow). PET, positron emission tomography

A 62-year-old male presented to the oncologist's office for evaluation of a progressively enlarging right submandibular swelling for seven months that was noted to have undergone significant enlargement in two weeks prior to presentation, now being larger than the size of a golf ball (Figure 1). The patient had already undergone evaluation of the mass by his primary care physician including a computed tomography (CT) of the neck (Figure 2), which showed a 1.8 cm solid nodule lateral to the right submandibular gland, a fine needle aspiration that demonstrated monomorphic population of lymphocytes consistent with malignant lymphoma, and a subsequent core biopsy (Figure 3) that was incidentally positive for a high-grade (grade 3) neuroendocrine carcinoma with a proliferative index greater than 60%. A positron emission tomography (PET) scan showed the hypermetabolic right facial soft tissue mass (4.5 x 3.9 cm with a maximum standardized uptake value [SUV] of 6.5) with hypermetabolic right cervical (level 2 and level 3 with SUV 6.2) lymphadenopathy (Figure 4). On physical examination, vital signs were stable. The right submandibular mass was large (about 5 cm), obvious, multiloculated, and associated with stretching of the overlying skin without any induration (Figure 1). At this point a decision was made to hospitalize the patient for further evaluation due to rapid enlargement of the mass. Laboratory evaluation revealed a white blood cell count of 6.69 x 10^3^/µL, hemoglobin of 9.8 g/dL, and platelet count of 273 x 10^3^/µL. Complete metabolic profile was essentially normal. During the hospitalization, reverse transcriptase-polymerase chain reaction test was performed on the previously performed core biopsy which confirmed group IIIA, stage 1, intermediate-risk alveolar RMS. The patient was treated with vincristine, actinomycin D, and cyclophosphamide (VAC) for intermediate-risk RMS. Definitive radiation therapy was also added to the patient's treatment regimen. She initially received a dose of 3,600 cGy in 20 fractions as external beam radiation therapy was then followed with a right neck boost for an additional 1,440 cGy in eight fractions to complete a total dose of 5,040 cGy. The patient initially responded to treatment with significant shrinkage and regression of the bulky tumor but unfortunately had recurrence after nine months with rapidly progressive tumor and distant metastasis that did not respond to salvage chemotherapy with vincristine, and irinotecan in addition to immunotherapy with regorafenib. He received additional palliative radiotherapy to manage local symptoms. The patient decided to opt for hospice. 

## Discussion

RMS is a soft tissue tumor arising from myogenic precursor cells occurring predominantly in children with most cases identified within first 10 years of life. In adults, RMS is very uncommon and highly malignant accounting for <1% of all solid tumor malignancies. Although common in the pediatric population, it differs in adult in terms of presentation, histological distribution, treatment, and outcomes [2]. Adults with RMS have a poorer prognosis than pediatric patients, and survival varies considerably depending on histological subtypes. RMS is most often sporadic, but certain cases are associated with familial syndromes such as Li-Fraumeni syndrome, neurofibromatosis type I, and hereditary retinoblastoma [3]. RMS can arise in a variety of anatomical sites with most common sites being head and neck (35%), pelvic region followed by extremities. Symptoms and clinical features of RMS vary depending on location, in our case as an example the patient presented with painless lump or swelling and facial asymmetry.

Histological subtypes of RMS comprise of embryonal (ERMS), alveolar (ARMS), pleomorphic (PRMS), and spindle cells/sclerosing types. Embryonal is the most common form overall [4]. ERMS and ARMS are the major subtypes seen in the pediatric population. ERMS tends to occur in younger children, and carries a better prognosis. ARMS occurs more frequently in adolescents and has more aggressive biological behavior [5]. PRMS occurs in both children and adults, but in adults it has very poor outcomes with higher rates of recurrence and metastasis [6,7]. Spindle cell RMS was traditionally included as a variant of ERMS, but it is now provisionally listed as a separate spindle cell/sclerosing RMS subtype in the latest World Health Organization (WHO) classification. Recently, a rare epithelioid variant of RMS mimicking carcinoma or melanoma that frequently occurs in older patients has also been described [8]. Here we present a case of alveolar subtype of RMS which represents 31% of all cases. In about 75% of RMS cases, chromosomal translocation results in the fusion of two transcription factor-encoding genes: the paired box gene 3 (PAX3), or less frequently the PAX7 gene and the Forkhead box protein O1 (FOXO1) gene. This fusion results in RMS tumor cells expressing chimeric PAX3/7-FOXO1 protein [9]. Important functions of tyrosine-protein kinase Met (MET) signaling in the development of RMS and its role in early metastasis have been described. Hence, in RMS, MET downregulation induces myogenic differentiation of tumor cells and as a consequence, metastatic potential of RMS cells is diminished. Therefore, blocking of MET may be clinically useful as a targeted therapy for RMS in future [10]. Common sites of metastases reported by conventional investigations are lungs, skeletal system, lymph nodes, and brain, with hematogenous being common route of metastases [11].

Diagnosing RMS is difficult with histology alone. Immunostaining is one of the most important methods that can lead to a specific diagnosis. Since our case demonstrated cell adhesion molecule (CD56) positivity, it may closely mimic a variety of other small round cell tumors, such as small cell carcinoma, lymphoma, neuroblastoma, and malignant melanoma. But further workup showed no immunoreaction to other solid tumor markers but was positive for myogenic differentiation 1 (MyoD1), desmin, and B-cell lymphoma 2 (BCL2). The histomorphology and immunohistochemical staining characteristics of the tumor cells were consistent with RMS with features of alveolar variant. Immunostains for MyoD1, desmin, BCL2, and CD56 were strongly positive in the tumor cells. The immunostain for chromogranin shows weak equivocal positivity, and anti-cytokeratin (CAM5.2) shows focal perinuclear punctate staining. The immunostain for mindbomb E3 ubiquitin protein ligase 1 decorates approximately 50% of the tumor cells. Other malignant tumors with rhabdomyosarcomatous differentiation need to be considered on the differential diagnosis. For example, malignant peripheral nerve sheath tumor with heterologous rhabdomyosarcomatous differentiation particularly in patients with history of type 1 neurofibromatosis. Immunohistochemistry for S100 and SRY-related HMG-box 10 can help to differentiate diagnostically [12].

CT and magnetic resonance imaging are used as a guidance to confirm the location, size, and infiltration of the tumor. A contrast-enhanced CT imaging is done to evaluate the primary tumor and further define the surrounding infiltration of vital structures as well as to look for any bone erosion. Metastatic evaluation includes bone scan and abdominal CT to determine the staging. A systematic review by Norman et al. concluded that fluorodeoxyglucose-PET/CT performed consistently better than conventional imaging in initial staging and restaging, and it has a potential role in assessing treatment response [13]. The definitive diagnosis of alveolar RMS requires histopathological examination. The histological appearance of alveolar RMS in our case was characterized by aggregates of small, round tumor cells, separated by fibrous septa. Microscopically, alveolar RMS is highly cellular, composed of primitive cells with monomorphous round nuclei and formed alveolar structures.

Treatment of RMS is based on risk stratification at the time of diagnosis. Surgical removal of the tumor and chemotherapy or combination of both is the treatment of choice. A complete resection is achieved by resecting the tumor along with 0.5 cm rim of normal tissue around it. Regional lymph node involvement in patients with alveolar RMS and distant metastasis is associated with a less favorable prognosis compared with those patients with localized disease. Radiotherapy is reserved only for patients who develop recurrence following completion of initial treatment. VAC is the most commonly used chemotherapeutic agent [14]. In cases of relapsing alveolar RMS, there is no consensus on second-line treatment; however, the combination of temozolomide and irinotecan has been considered a possible option [15]. In recent years, the use of brachytherapy in head and neck tumors has become increasingly popular owing to its superiority in tissue-sparing approach. New studies are evaluating the use of immunotherapy like regorafenib in advanced sarcomas [16].

According to the Intergroup Rhabdomyosarcoma Study Group (IRSG), surgicopathological staging of RMS is predictive of outcome [17]. Some data indicate that staining for myogenin correlates with decreased survival [18]. Some studies also reported that anatomic site was also a significant prognostic indicator [19]. The survival of adult populations with RMS is low with five-year overall survival rates ranging from 40% to 54%. Some of this difference in outcome between children and adults has been attributed to increased incidence of poor prognostic features in adults such as unfavorable primary site, unfavorable histology, and higher rates of regional and distant spread [20].

## Conclusions

Neck masses can represent a variety of benign to highly malignant disease processes. A careful history and physical exam is essential for diagnosis. Biopsy for histopathological examination is also necessary for correct diagnosis. Treatment when tailored according to correct diagnosis can lead to improvement in quality of life and sometimes survival. Although rare, RMS should fall in the list of differentials for older patients as well.
